# A mixed method study of menstrual health in Spain: pain, disorders, and the journey for health

**DOI:** 10.3389/fpubh.2025.1517302

**Published:** 2025-02-25

**Authors:** Sara Sánchez-López, Dani Jennifer Barrington, Rocio Poveda-Bautista, Santiago Moll-López

**Affiliations:** ^1^INGENIO (CSIC-UPV), Universitat Politècnica de València, Valencia, Spain; ^2^School of Population and Global Health, University of Western Australia, Crawley, WA, Australia; ^3^Departamento de Matemática Aplicada, Universitat Politècnica de València, Valencia, Spain

**Keywords:** menstrual health, dysmenorrhea, healthcare access, gender pain gap, health inequalities

## Abstract

**Introduction:**

Menstrual-related symptoms and disorders, particularly dysmenorrhea, significantly impact women's well-being. Dysmenorrhea, marked by painful menstrual cramps, affects up to 95% of women, leading to debilitating symptoms that interfere with daily activities and potentially signal underlying gynecological issues. Despite its prevalence, many women experience inadequate medical support and dismissive healthcare responses.

**Methods:**

This study employed a mixed-method approach, combining quantitative and qualitative survey data to explore Spanish women's experiences with menstrual discomforts and healthcare access. A total of 3,490 participants responded to the survey, which assessed the prevalence of menstrual discomforts, frequency of medical consultations, and perceived quality of gynecological care.

**Results:**

Findings indicate that 70.9% of participants experience menstrual discomforts monthly or most months; despite this, a significant number of women infrequently seek gynecological care, often due to perceived dismissiveness and inadequate medical support. The qualitative analysis reflects shared experiences of pain normalization, misattribution to other conditions, and dissatisfaction with the standard treatment of contraceptive pills without thorough diagnostics. Additionally, the study reveals that in Spain, access to healthcare support for menstrual issues is highly influenced by whether the provider is public or private, demonstrating the impact of socio-economic factors and underscoring a core contributor to health inequity.

**Discussion:**

This study highlights the persistent gender pain gap and the need for a more holistic and empathetic approach in medical practices. The authors' recommendations include incorporating gender training for healthcare professionals and promoting awareness campaigns to encourage medical consultations for menstrual pain. This research aims to improve support systems and healthcare practices, enhancing the quality of life for women in Spain.

## 1 Introduction

Menstrual-related symptoms and disorders represent an important yet often under-recognized issue affecting women's daily lives. Dysmenorrhea, the most common menstrual-related condition, is defined as painful menstrual cramps of uterine origin, and it is characterized by abdominal pain before or during menstruation (1). It may affect up to 95% of women and people who menstruate, and is prevalent across brackets of socioeconomic status, ethnicity, and nationality (2, 3). Dysmenorrhea itself can cause debilitating symptoms and have a significant impact on overall physical, mental and social health; it might also indicate several gynecological disorders or be a symptom of other underlying health issues (3).

Dysmenorrhea can be classified into two forms: primary and secondary. Primary dysmenorrhea occurs without any underlying pathology and is typically linked to uterine contractions caused by prostaglandin release. In contrast, secondary dysmenorrhea arises from gynecological underlying conditions like endometriosis (2, 4). Endometriosis is a gynecological condition characterized by the presence of endometrial tissue outside the uterus, while adenomyosis is a hormone-related disorder defined by the infiltration of endometrial glands and stroma into the myometrium (5). Both present significant diagnostic challenges, partly because both conditions have non-specific manifestations, with symptoms such as chronic pelvic pain, heavy menstrual bleeding, and severe dysmenorrhea overlapping with other pathologies (6). These overlaps frequently result in misdiagnosis or delayed diagnosis, particularly in adolescents and young women (5). Failing to address the symptoms of primary dysmenorrhea not only misses an opportunity to improve the patient's quality of life but also delays the diagnosis of underlying conditions like endometriosis and adenomyosis.

Dysmenorrhea is one of several symptoms which may be menstrual-related but are often overlooked. These can range from gastrointestinal symptoms like vomiting and diarrhea, to systemic effects such as abdominal swelling (bloating), pain, and heavy bleeding, all contributing to menstrual distress (7–9). The repercussions of these symptoms are far-reaching and can reduce the quality of life, interfering with daily activities and leading to absenteeism from school, work, and social activities (3, 10–12). Chronic gynecological conditions associated with secondary dysmenorrhea, such as endometriosis or adenomyosis, may also increase the risk of developing chronic pelvic pain syndromes and infertility if left untreated (4). There is also a correlation between dysmenorrhea and other chronic pain conditions such as migraine, fibromyalgia, and irritable bowel syndrome, which suggests a heightened pain sensitivity, and raises the potential for an increased risk of chronic pain syndromes later in life for these women (8–10, 13).

Thus, the support—or lack thereof—that women receive in the context of seeking relief from menstrual discomforts plays a pivotal role in their management strategies and overall health outcomes. The journey followed by individuals to restore their health, from the first signs of symptoms until the confirmation of the diagnosis and the provision of suitable treatment, is known as a *therapeutic itinerary* (14). This path is influenced by socio-cultural practices, social context, economic factors (15), and gender. The *gender pain gap* refers to the differences in how pain is perceived, treated, and responded to between genders in medical settings (16). It primarily highlights how women's pain is often under-recognized and undertreated compared to men's, influenced by biases in medical research and healthcare practices (17), which hinders the provision of adequate health solutions for women (18). Comprehending the challenges encountered in the search for support is a valuable tool for the improvement of health assistance (19).

This research uses a Spanish case study to explore women's perceptions and experiences in addressing these matters. Understanding the health context of Spain is crucial since it directly impacts women's access to care and their overall health outcomes. In the Spanish public health system, one needs a referral from a family doctor to see a specialist, and family doctors may not grant one if they deem it unnecessary. Access to gynecological care can be limited due to high demand and limited resources, leading to long waiting times (averaging 72 days for the first consultation in 2023) (20) and potential delays in diagnosis and treatment. In 2021, in Spain, there were 6,166 obstetricians and gynecologists (21), and a population of 22,138,643 women over the age of 10 (22), resulting in an average of 3,590 people per professional. Long wait times for appointments, tests, results, and subsequent treatment and follow-up can stretch over years, further driving individuals toward private care, as evidenced by the vast difference in the number of gynecological consultations between the public system (26,642) and the private system (493,431) during 2021 (23).

This study aims to explore women's experiences relating to menstrual discomforts and their experiences accessing healthcare for menstrual-related issues in Spain using a mixed approach of qualitative and quantitative methods, building on a previous work that examined menstrual literacy and experiences within the Spanish context (24). The objective is to advocate for improved support systems and healthcare practices that validate and respond to women's needs, ultimately enhancing their quality of life.

## 2 Materials and methods

This research adopted a constructivist/interpretivist paradigm, which views knowledge and reality as constructed through social interactions and interpreted based on individual experiences and cultural contexts (25). By focusing on the Spanish setting, this approach enabled the researchers to examine how various factors, such as gender, socioeconomic status, and cultural norms, shape women's health support access.

For clarity and transparency, this paper adheres to the Standards for Reporting Qualitative Research (SRQR) (26) as recommended by the EQUATOR Network (27) (see Appendix).

### 2.1 Participants and recruitment

A comprehensive and exploratory survey on menstruation was conducted in Spain over a period of nine months (May 2021–January 2022). The target demographic population included individuals over 14 years old, of all sexes and genders (many questions were relevant to both those who do and do not menstruate), and who were either native to Spain or residing in Spain and its territories. The survey was disseminated through a varied distribution approach that combined convenience sampling via WhatsApp groups, social media platforms (Instagram, Facebook, and Twitter), and snowball sampling.

A total of 4,028 participants took part. The survey was designed to target a diverse age demographic, successfully gathering a sample reflecting the age distribution of Spain. Participation spanned from an initial 0.01% for those born in the decade of the 2000s, increasing up to 0.06% for the 1990s, and covering significant representation across all other age segments. Recognizing the potential bias introduced by the digital format of the survey toward younger participants, older age groups were specifically targeted to secure a significant minimum representation of these demographics in our study. For the present paper, only the answers of those who currently menstruate or who had menstruated in the past, were used.

Additionally, the survey aimed to provide an extensive geographic representation and capture the demographic characteristics of each Spanish region. When conducting our survey, the demographics of the responses were monitored, and additional efforts were made to circulate the survey within those areas with less representation until adequate representation was achieved from each region in proportion to their population size.

### 2.2 Data collection instrument. Survey design

The survey tool was developed considering insights from prior studies (24, 28–30). Administered through the digital platform Typeform, the questionnaire included quantitative and qualitative elements to ensure a comprehensive understanding of the wide range of perceptions and experiences associated with menstruation in Spain. It included 43 items, spanning single and multiple-choice questions, rating Likert scales, dropdown selections, and open-ended responses to facilitate comprehensive insights into menstrual experiences and perceptions in Spain. It concluded with the question, “Please use this space to share any doubts, comments, or reflections on this topic. You can also share experiences or anecdotes related to menstruation.”

The online survey was designed to be user-friendly and clear for everyone, employing simple and plain language. This strategy was designed to maximize the diversity and representativeness of the respondent pool, facilitating engagement from a broad segment of the Spanish population across a wide age range and socio-economic statuses. The questionnaire's research areas included *demographic data, menstrual information* for biological and educational insights, *menstrual healthcare* for assessing healthcare interactions, *menstrual discomforts* to identify common issues, and an open-ended question for personal narratives.

The survey included logic that adapted questions based on the previously selected answers; however, all participants were asked a final open-ended question. Following a pilot study with 45 individuals and refinements based on expert feedback, the survey's reliability was confirmed by a Cronbach's alpha of 0.81.

### 2.3 A note on gender terminology

The authors recognize and respect the diversity in gender identities. This particular study reflects on how personal perceptions of pain and interactions with healthcare professionals may be influenced by traditional narratives and ideologies surrounding womanhood, as well as the patriarchal system. Consequently, in this paper, the term *women* will be used to refer specifically to cisgender women. We arrived at this decision because our qualitative data primarily includes responses from individuals who menstruate and identify as women (we know this due to the gendered nature of the Spanish language). Although our quantitative data do include transgender respondents, their experiences represent a different context and set of challenges that are not reflected in our qualitative findings. Therefore, we will speak through the lens of cisgender women to accurately reflect the data discussed in this study, but note that there is a pressing need to understand the experiences of those who menstruate but do not identify as women.

### 2.4 Data analysis

The approach to data analysis combined descriptive and inferential statistics (SPSS) as well as qualitative coding (Nvivo 12) to delve into the perceptions and experiences of menstruation. Demographic details were reported using frequencies and percentages. For the quantitative data, the Shapiro-Wilk test, used to assess the normality of questionnaire scores, revealed a non-normal distribution for many of the scores. Consequently, both parametric (where applicable) and non-parametric statistical methods were employed. The latter included the independent samples Kruskal-Wallis test and the Mann-Whitney *U* test for instances lacking normal distribution. The Chi-square test was used to investigate relationships between qualitative variables, and linear regression and the Student *t*-test were utilized to explore correlations and dependencies among variables.

The final, open-ended question was analyzed thematically. This facilitated an in-depth exploration of significant menstrual health topics as articulated by respondents. For the present paper, only the narrative responses that detailed menstrual discomforts are discussed.

This blend of quantitative and qualitative analysis methods, processed through SPSS software and Nvivo 12, enabled a robust understanding of menstrual experiences and the healthcare dynamics for matters related to menstruation within Spain, grounding the study in a comprehensive analytical framework.

### 2.5 Reflexivity

Throughout the study, reflexivity was practiced to ensure a rigorous understanding and acknowledgment of the researchers' influence on the research process and its outcomes. Reflexivity involves recognizing and critically evaluating the effect of the researchers' personal beliefs, values, and experiences on all aspects of the research, from data gathering and analysis to the interpretation of the outcomes (31). Researchers for the present study are white cisgender women and one cisgender man from Spain and Australia, with experience in social research.

The research team engaged in regular discussions to foster collective reflexivity. These discussions provided a platform for challenging and scrutinizing each other's assumptions and methodological choices, enriching the research process with diverse viewpoints. Peer debriefing sessions involved discussions with colleagues with different backgrounds, including sociologists, gender researchers, political scientists, data scientists, and engineers. These sessions supported reflective practice and offered external perspectives that prompted additional reflexivity. Together, these strategies reinforced the study's commitment to mitigate bias and enhance the integrity of the research.

## 3 Results

### 3.1 Menstrual discomforts

This section of the study explores the prevalence and types of menstrual discomfort experienced by the participants. Participants who menstruate/d were asked whether they experienced discomfort and what kind, allowing multiple selections for the types of discomfort experienced. Only 2.5% reported never experiencing any menstrual discomfort, whereas 42.5% encountered it consistently every month, 28.4% faced discomfort most months, and 26.6% experienced it occasionally (see Table 1). That is, 70.9% of the respondents reported experiencing menstrual discomforts either every month or most months.

**Table 1:** Frequency and type of menstrual discomforts.

**Discomfort**	**Total**	**Percentage (%)**
Pain	2,968	84.4
Vomiting	243	6.9
Diarrhea	1,808	51.4
Abdominal swelling	2,617	74.5
Particularly heavy bleeding	1,520	43.2
None	122	3.5
**Frequency of any discomfort**		
Every month	1,477	42.5
Most months	987	28.4
Some months	924	26.6
Never	86	2.5

Pain was reported by 84.4%, while 74.5% experienced abdominal swelling, 51.4% diarrhea, and 43.2% dealt with heavy bleeding. Other frequently cited symptoms included headache, cramps, insomnia, breast pain, nausea, lower back pain, anemia, fatigue, increased hunger, and mood swings.

Table 2 shows the percentages of menstrual discomfort across different decades of birth. As the birth year moves toward the 2000s, the percentage of respondents experiencing discomfort *every month* increases. The results of the Chi-square tests, with a significant *p*-value (*p* < 0.001), indicate that the differences in discomfort frequency across the decades are not due to chance.

**Table 2:** Decade of birth vs. frequency of menstrual discomforts.

**Decade of birth**	**Every month**	**Most months**	**Some months**	**Never**
1950	37.5%	15.6%	37.5%	9.4%
1960	26.2%	29.7%	40.0%	4.1%
1970	34.4%	32.1%	30.7%	2.8%
1980	41.5%	27.5%	28.8%	2.1%
1990	47.3%	28.0%	22.5%	2.2%
2000	48.7%	27.5%	21.4%	2.3%

Percentages calculated per decade.

The data in Table 3 shows that over the decades from the 1950s to the 2000s, more women reported experiencing menstrual discomforts such as pain and abdominal inflammation. Specifically, the reports of pain rose significantly from 57.1% to 90.4%, and reports of abdominal inflammation went from 60.0% to 72.2%. There has also been an increase in complaints of diarrhea and intense bleeding, while fewer women are reporting no discomfort at all—dropping from 5.7% to 4.3%. The symptoms specified here are tabulated without frequency. Overall, the differences observed across the decades are significant and highlight a potential trend of increasing menstrual discomforts over time.

**Table 3:** Decade of birth against menstrual discomforts.

**Decade of birth**	**Pain**	**Abd. inflammation**	**Diarrhea**	**Intense bleeding**	**Vomiting**	**Nothing**
1950	57.1%	60.0%	11.4%	34.3%	8.6%	5.7%
1960	65.8%	62.3%	28.1%	52.1%	6.2%	5.5%
1970	77.3%	73.7%	39.2%	50.2%	5.7%	5.5%
1980	84.9%	72.2%	48.7%	41.6%	5.0%	2.9%
1990	88.7%	79.4%	61.9%	39.5%	8.0%	2.4%
2000	90.4%	72.2%	55.9%	49.0%	11.0%	4.3%

Multiple choice allowed. Percentages calculated per decade.

### 3.2 Medical support

Participants who menstruate/d were asked how often they attended a gynecological consultation. The data obtained (Table 4) show that 39.1% of the participants visit a gynecologist annually. However, 35.6% of respondents reported visiting less than once a year, and 19% never visited at all. Those who attend more frequently, either twice a year or more than twice a year, account for relatively small percentages, 4.4% and 1.9%, respectively.

**Table 4:** Frequency of gynecologist consultations.

**How often do you consult a gynecologist?**	**Frequency**	**Percent**
Never	662	19.0%
Less than once a year	1,237	35.6%
Once a year	1,361	39.1%
Twice a year	153	4.4%
More than twice a year	66	1.9%
Total women	3,479	100.0%

To explore the reasons behind these consultation frequencies, we examined the relationship between the frequency of gynecological visits and the prevalence of menstrual discomforts.

The pattern observed in Table 5 indicates that visits to the gynecologist *Twice a year or more* are rare across all frequencies of menstrual discomfort. For women experiencing discomfort *every month*, only 5.3% visit *twice a year*, and just 2.2% visit *more than twice a year*. Similarly, for those with discomfort *most months*, only 4.3% visit *twice a year*, and 1.1% visit *more than twice a year*.

**Table 5:** Percentages of respondents' frequency of menstrual discomfort vs. frequency of visits to the gynecologist.

	**Frequency of visits to the gynecologist**				
**Frequency discomforts**	**Never**	**Less than once a year**	**Once a year**	**Twice a year**	**More than twice a year**
Never	26.7%	30.2%	40.7%	0.0%	2.3%
Some months	16.9%	37.4%	40.0%	3.5%	2.3%
Most months	18.5%	37.4%	38.7%	4.3%	1.1%
Every month	20.3%	33.6%	38.7%	5.3%	2.2%

Notably, 20.3% of the respondents experiencing menstrual discomforts *every month* have never sought gynecological support. The distribution is more balanced for those with occasional discomfort, with the majority (40.0%) visiting once a year.

These findings suggest that while regular discomforts generally lead to more frequent gynecological consultations, high-frequency visits are uncommon. The data may highlight a significant gap in regular medical consultations for menstrual issues (or discomforts), pointing to potential barriers to accessing frequent gynecological care. Additionally, a considerable number of women, even those experiencing regular discomfort, may not be seeking medical support.

A Chi-square test was conducted to examine the association between the frequency of menstrual discomforts and the frequency of visits to the gynecologist. The significance value of 0.025 suggests that there are differences in how often women visit the gynecologist based on their experience of menstrual discomforts, despite not following a simple linear pattern.

Pearson and Spearman correlation coefficients revealed shallow correlation values (Pearson: *p* = 0.006, Spearman: *p* = 0.004) with high *p*-values (Pearson: *p* = 0.725, Spearman: *p* = 0.824), indicating no significant statistical correlation between the frequency of menstrual discomforts and the frequency of gynecologist visits. This lack of correlation suggests that other factors, like menstrual literacy, accessibility to healthcare, normalization of menstrual-related symptoms or personal preferences, may influence how often women visit a gynecologist.

#### 3.2.1 The use of public or private health system

Considering that there may be other factors that can influence the frequency of consulting a gynecologist, a Chi-square test and a crosstabulation of three variables were conducted attending to the frequency of menstrual discomfort, the frequency of gynecologist visits, and whether the healthcare provider was private or public. The Pearson Chi-square test shows a very low *p*-value (0.001) when considering the entire dataset, indicating a statistically significant association between the frequency of menstrual discomfort, gynecologist visits, and the type of healthcare provider (private vs. public).

When the type of healthcare provider separates the data, the Chi-square test shows a significant association for private healthcare (*p*-value = 0.041) but not for public healthcare (*p*-value = 0.313). This suggests that private healthcare users show an association between menstrual discomfort and visit frequency, while public healthcare users do not. It is possible that private healthcare users are more likely to visit the gynecologist in response to menstrual discomfort, or it could be due to factors such as availability or scheduling flexibility.

**Figure 1:**
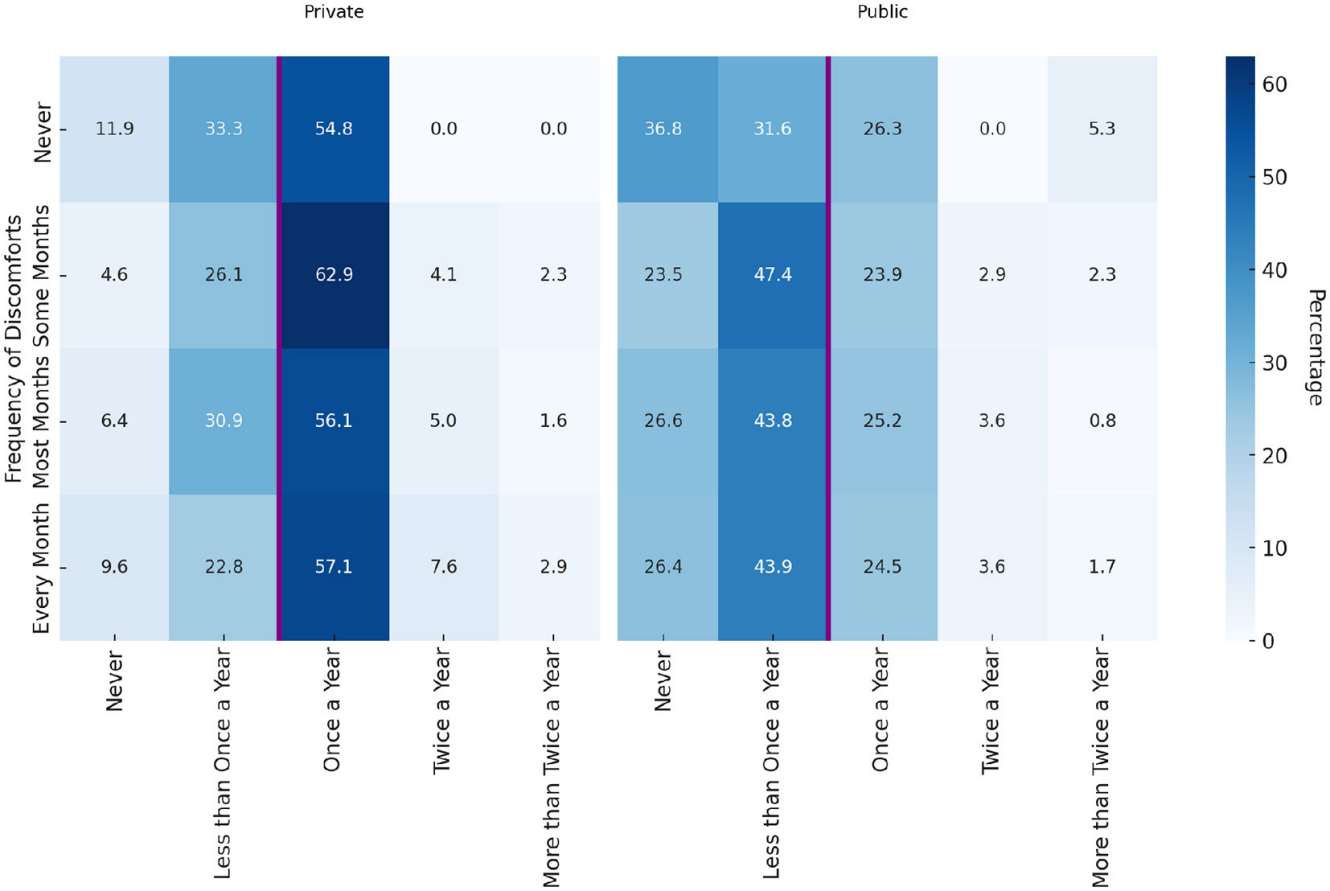
Row percentages of gynecologist visits by menstrual discomfort frequency in private and public settings.

Individuals who experience menstrual discomfort *some months* or *most months* tend to visit *less than once a year* more frequently in public healthcare (47.4% and 43.8%, respectively) than in private healthcare (26.1% and 30.9%, respectively) (see Figure 1). On the other hand, high-frequency visits are relatively rare in both settings but slightly more common in private healthcare, with *every month* discomforts resulting in 2.9% visiting *more than twice a year* compared to 1.7% in public healthcare.

Based on the data in Figure 1, women who experience menstrual discomfort more frequently, particularly *every month*, do not necessarily visit the gynecologist more often than those with less frequent discomfort. It appears that those experiencing discomfort *some months* or *most months* have higher visit frequencies. It may be easier to identify that something might be wrong or abnormal when discomforts are present in all their periods than when the periods and discomforts are always together. This normalization of pain might explain why those with monthly discomfort do not show significantly higher visit rates.

In both private and public settings, across all frequencies of visits, most visits to the gynecologist were made by women in their menstrual and peri-menopausal phases. Interestingly, while the overall frequency of visits by post-menopausal women is low in both settings, private settings see a higher percentage of annual visits (Table 6). On the contrary, a higher percentage of women using public settings have visited less than once a year or never. This difference could indicate barriers to access or different health-seeking behaviors in public healthcare systems.

**Table 6:** Row percentages of gynecologist visits in private and public settings by menstrual maturation phase.

**Reproductive phase**	**Private**					**Public**				
	**More than twice a year**	**Twice a year**	**Once a year**	**Less than once a year**	**Never**	**More than twice a year**	**Twice a year**	**Once a year**	**Less than once a year**	**Never**
Menstruation and peri-menopause	2.2%	5.9%	57.2%	26.9%	7.7%	1.6%	3.1%	23.5%	44.6%	27.2%
Post menopause	2.9%	4.8%	71.4%	17.1%	3.8%	2.3%	6.0%	39.1%	44.4%	8.3%

There is a difference in the frequency of gynecological visits between private and public healthcare. The distribution of visits in public healthcare is skewed toward less frequent visits (*less than once a year* and *never*), whereas private healthcare users seem to adhere more to a more frequent pattern (*once a year, twice a year*, and *more than twice a year*). The majority of those who menstruate or have menstruated tend to visit the gynecologist once a year, with a significantly higher percentage observed for women who are post-menopause.

Most women who are post-menopause and using private healthcare visit the gynecologist once a year (71.4%, see Table 6), whereas the frequency is lower for those using public healthcare. This could indicate that interest in medical support during post-menopause exists, but it is easier to obtain for private health users. It might also be related to the demographic difference among public and private health users.

#### 3.2.2 Relation with income

Different statistical analyses were conducted to analyse the potential relationship between monthly income and healthcare provider choice. Chi-square test results indicate significant associations (*p* < 0.001) in monthly income distribution between private and public healthcare users. The data shown in Table 7 suggest that individuals' income levels may be a determining factor in their choice of healthcare provider. Similarly, the logistic regression analysis shows a statistically significant association between income and the likelihood of choosing private healthcare over public: as income increases, the likelihood of opting for private healthcare also increases. However, given the small R-squared values, income alone does not account for most of the variability in the healthcare provider choice.

**Table 7:** Distribution of monthly income in relation to healthcare provider choice.

**Monthly income**	**Private**	**Public**
No income	38.4%	61.6%
1 to 400 €	33.3%	66.7%
400 to 800 €	37.6%	62.4%
800 to 1,200 €	38.6%	61.4%
1,200 to 1,600 €	46.9%	53.1%
1,600 to 2,000 €	57.0%	43.0%
2,000 to 2,500 €	61.9%	38.1%
More than 2,500 €	58.5%	41.5%
Prefer not to answer	43.8%	56.2%

#### 3.2.3 Decade of birth vs. frequency of visiting a gynecologist

To understand how the frequency of gynecologist visits varies across different birth groups, we analyzed the data by decade of birth. Results can be seen in Table 8.

**Table 8:** Frequency of gynecologist visits by birth decade.

**Decade of birth**	**More than twice a year**	**Twice a year**	**Once a year**	**Less than once a year**	**Never**
1950	0.0%	2.9%	62.9%	20.0%	14.3%
1960	2.7%	4.8%	54.1%	36.3%	2.1%
1970	2.8%	5.1%	45.6%	38.7%	7.8%
1980	2.1%	5.5%	46.7%	37.6%	8.0%
1990	1.5%	3.5%	32.8%	36.8%	25.4%
2000	1.2%	3.2%	19.0%	20.8%	55.8%

There appears to be a decrease in the percentage of people visiting the gynecologist *once a year* as we move from the 1950s cohort to the 2000s interval. The rate of individuals who have never visited a gynecologist increased significantly in the 1990s and 2000s compared to earlier decades. Visits to the gynecologist twice a year remained relatively stable through the 1960s to 1980s. Very few people visit the gynecologist more than twice a year across all decades, with a slight decrease over time.

The Pearson Chi-square (*p*-value) is less than 0.001, indicating that the patterns observed in the table are unlikely to have occurred by chance. This suggests a significant association between the decade of birth and gynecologist visit frequency, possibly due to women waiting until they are older to see the gynecologist.

### 3.3 Qualitative study

A total of 1,165 respondents (28.9%) answered the final open-ended question. Although no explicit question was posed to participants about medical support for menstrual-related issues, apart from inquiries regarding the frequency of their consultations with a gynecologist, the topic of doctor-patient interactions emerged prominently in responses to this question.

Participants frequently used this opportunity to express difficulties in obtaining professional support for menstrual disorders. Many reported their concerns being dismissed or inadequately handled by healthcare providers, highlighting medical support as a notable concern.

**Figure 2:**
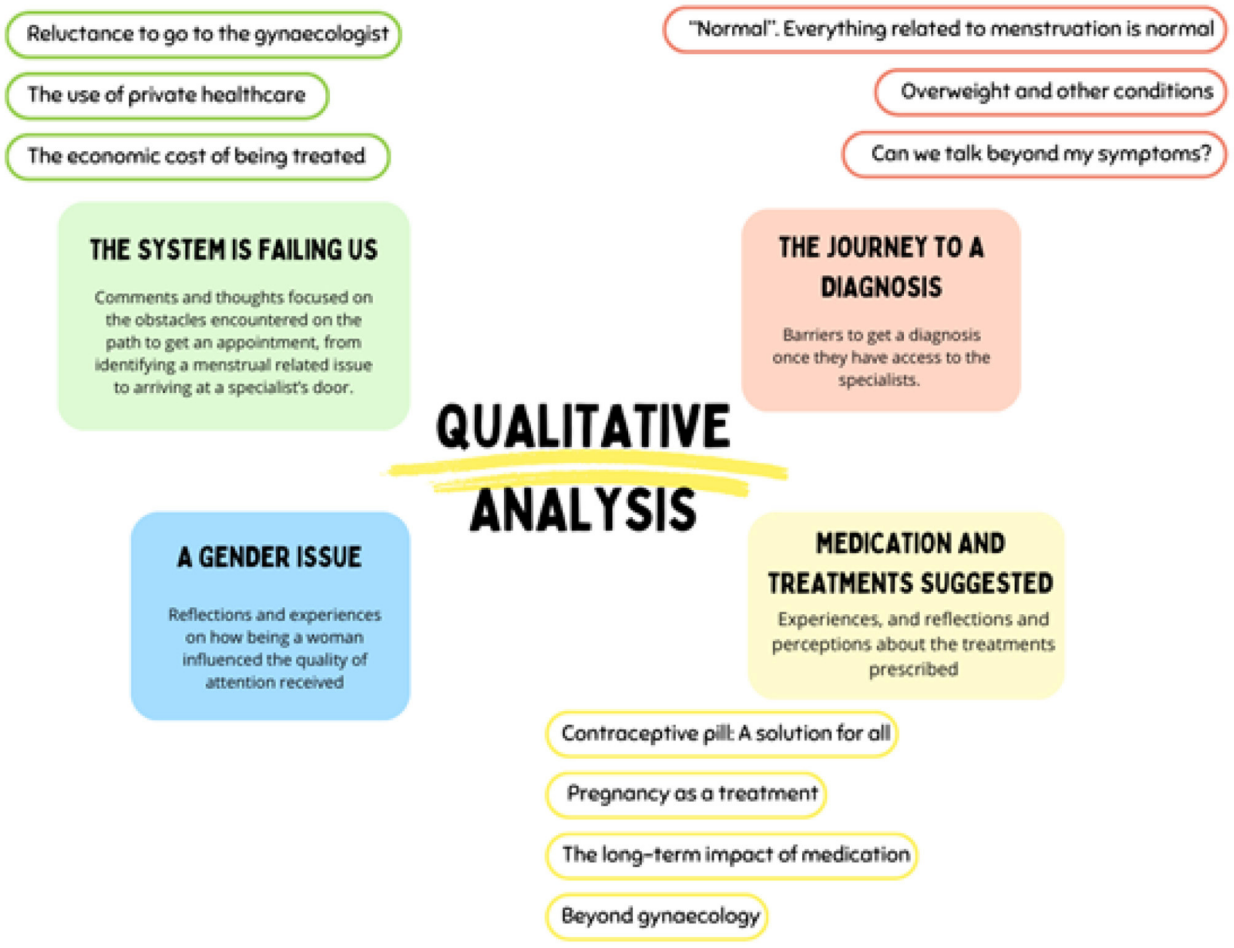
Themes related to menstrual discomforts identified during the qualitative analysis.

Four themes are identified from the qualitative analysis of the data (Figure 2). These are presented below and supported with illustrative quotations.

#### 3.3.1 The journey to diagnosis

“It's normal. The pain, the excessive bleeding. Everything related to menstruation is normal.”

A high number of respondents shared experiences where the doctors normalized their pain, excessive bleeding, or other discomforts. Often, respondents reported feeling dismissed and as if their pain was invalidated.

*Since I started menstruating, I have been experiencing horrible pains accompanied by vomiting, diarrhea, and dizziness. And yet, from the professional area, they still tell me it's normal*. (P3855, Madrid, YOB 1988)

While some respondents had accepted pain as part of menstruation and asked if it was true that periods do not need to be painful, many respondents criticized the idea of accepting pain as normal and pointed out the lack of support. The level of pain with which some respondents reported dealing was critical to the point of fainting or going to emergency medical care.

*...during my labor, I didn't ask for an epidural (12 h) since I usually treated those pains with ibuprofen, took the subway, and spent 12 h working*. (P2726, Madrid, YOB 1978)

Some participants perceived that asking for assistance with anomalies related to menstruation irritated doctors and was considered a waste of resources and time. Different testimonies described being accused of overrating their pain, or even outright lying about their symptoms. As a result, in addition to not being helped, individuals felt guilty or doubted that their pain was justification enough to seek medical support.

*... I have had to go to the emergency room on more than one occasion. From sexist comments to suggestions that I was making up symptoms (exaggerating or outright lying) to the most common issue, underestimating the problem*. (P1676, Galicia, YOB 1984)

#### 3.3.2 Overweight and other conditions

When other conditions were present, participants found that their symptoms were often attributed to these, which were used as a reason to disregard their symptoms. Overweight respondents reported difficulties in getting doctors to look beyond their weight to consider other potential causes. The experience was similar for respondents with a history of anxiety, who perceived not being taken seriously and felt their mental health history reduced their credibility. In addition to the frustration due to the lack of help, these interactions left the respondents feeling mistreated and disrespected.

*Since I started going to the gynecologist, they were always very unpleasant and their relationship with me was not exactly the most respectful. I have always been overweight, and that was the only thing they focused on: the root of all my evils. No matter the intensity of my pains or the irregularity of my menstruations, everything was about being overweight*. (P1029, Galicia, YOB 1996)

#### 3.3.3 Can we talk beyond my symptoms?

While pain and other discomforts were the primary reasons for seeking medical care, numerous respondents expressed the need to investigate potential underlying pathologies signaled by the described symptoms. The study gathered numerous accounts of how symptoms were either overlooked or treated with standard medication without examining the potential underlying cause. Years later, these individuals discovered pathologies that explained their symptoms.

*We take hormones, painkillers, anything that helps us cope better, but these are always solutions to the symptoms*. (P3269, Andaluía, YOB 1980)

Many participants narrate battling for years before being diagnosed despite the many attempts to get medical support from different doctors due to the extreme pain. They often expressed feelings that their concerns about an underlying pathology were disregarded and their pain and experiences were invalidated. As a result, many were misdiagnosed or not treated, extending their suffering for years or decades. In addition, in some cases, the lack of diagnosis and treatment worsened the condition over time.

*Apparently, that and other gynecological symptoms I've had were symptoms due to an autoimmune disease diagnosed later. The explanations and questions I posed to healthcare professionals were of no use to me*. (P1222, País Vasco, YOB 1981)

*It took 14 years for me to be diagnosed with endometriosis, by then I was already filled with endometriomas, and I couldn't even walk, but they said that menstrual pain was normal and to be expected*. (P1640, Castilla-La Mancha, YOB 1982)

#### 3.3.4 Medication and treatments suggested

##### 3.3.4.1 Contraceptive pill: a solution for all

Contraceptive pills were described by the respondents as the first and frequently the “*catch-all treatment for all the issues*” (P1102, Andalucia, YOB 1998) including pain, acne, or irregular bleeding, regardless of the individual's specific condition.

*...once a month I am not even able to get out of bed and the only solution gynecologists offer me are birth control pills. These, apart from making me feel terrible physically and emotionally and representing an extra expense, do not relieve my pains*. (P815, Extremadura, YOB 1995)

Some respondents reported an improvement in their quality of life when the contraception pill or the Intrauterine Device (IUD) was prescribed to them and decreased their pain. Yet, often, individuals expressed the desire for different alternatives. Participants also expressed concerns that these treatments, particularly contraceptive pills, were prescribed to them without prior exploration or analysis and that the potential secondary effects of this medication were not explained to them. Furthermore, if the patient declined the proposed contraceptive pill treatment, their reports of pain were questioned “*it must not be that painful then!*”- (P2597, Cataluña, YOB 1989) when quoting doctors. There were cases reported where hormonal treatments caused adverse effects, and when explained, these symptoms were minimized by doctors who did not provide alternative treatments.

*After my first menstruation, I did not menstruate for a year, and then it was tremendously irregular. This, along with the fact that I started to suffer from significant hair loss, led me to the gynecologist who prescribed me the pill at 17 without even performing a cytology or analysis. This caused a lot of side effects, which were not addressed because “it was normal.” Years later and after several visits, it wasn't until I changed gynecologists that one explained to me what PCOS (Polycystic Ovary Syndrome) was*. (P879 Madrid, YOB 1995)

##### 3.3.4.2 Pregnancy as a treatment

Pregnancy was suggested to many respondents as a solution for abnormal bleeding and painful menstruation. In some cases, it was assumed that the patient would have children in the future, and that would solve the problem. Participants commented on this instrumentalization of pregnancy as a treatment with irony and disbelief.

*...the solution doctors gave me was to have a child, and then my menstrual pains would go away*. (P753, Andalucia, YOB 1982)

*...the doctor's response is “it will sort itself out when you have children.”* (P3874, Castilla La Mancha, YOB, 1986)

*I have been diagnosed with endometriosis and am finding it very difficult to receive appropriate treatment. Doctors have told me to get pregnant*. (P2904, Comunidad Valenciana, YOB 1982)

##### 3.3.4.3 Concern about the future: the long-term impact of medication

The consumption of medication to deal with menstrual pain was described in the comments as a common practice. Some participants voiced concerns over the long-term implications of regularly using medications like contraceptive pills and pain relievers, e.g., Ibuprofen.

*That medication (hormones or pain relievers) has such a list of side effects that it seems as if no one, neither pharmaceutical companies, governments, nor society in general, cares about the future problems they may be causing us*. (P3125, Rumania, YOB 1994)

##### 3.3.4.4 Beyond gynecology

Some participants emphasized the significant improvement in quality of life they experienced when they received accurate diagnoses, often, highlighting the need to consult beyond gynecology. This was in line with the desire voiced in the responses for a more holistic perspective to treat their discomforts, including other areas of expertise. The most named areas among the respondents were endocrinologists, pelvic floor physiotherapists, and nutritionists.

#### 3.3.5 The system is failing us

##### 3.3.5.1 Reluctance to go to the gynecologist

Many participants were hesitant to visit a gynecologist for a variety of reasons. These included not trusting doctors, fear of mistreatment, feeling unheard or ignored, or concerns about physical discomfort during the exploration. Others doubted the doctors' expertise or their willingness to offer help beyond standard medications.

*I recognize that I no longer go to the gynecologist because whenever I have gone, I always felt that I was treated poorly or my pains were belittled*. (P1561, Madrid, YOB 1989)

*Gynecologists, in general, lack empathy and information*. (P1379, Andalucia, YOB 1980)

Respondents often believed that going to the gynecologist would not be helpful and felt it was not worth the effort, time, and/or money. They did not trust that they would be listened to and examined to find the cause of the problems, or given alternatives. Some of these reasons came from their own bad experiences, while others were influenced by stories shared by other people.

*I find it easier and more enriching to talk with other women about menstruation than with doctors*. (P1919, Cantabria, 1980)

*I think a lot about the aversion I feel toward going to the gynecologist, fearing that they will prescribe me the pill without conducting a thorough diagnosis and without proposing other solutions, as I see is common among people I know*. (P1267, Castilla y León, YOB 2001)

##### 3.3.5.2 Private health

Despite the expense, many respondents who can afford it choose private doctors. Several reasons were highlighted for this preference.

Accessing gynecologists via public healthcare in Spain is not necessarily straightforward or swift. Long wait times for appointments, tests, results, and subsequent treatment and follow-up can stretch over the years, further driving individuals toward private care. Some participants narrated being denied the possibility of being referred to a public gynecologist, and others commented on the challenges of getting a follow-up appointment to check the prescribed treatment.

*Every time I've mentioned to my primary care physician about referring me to a gynecologist, I've been told, “why bother if you're young and all girls have painful menstruations.”* (P1388, Islas Canarias, YOB 1998)

The lack of solutions provided to them by the public health system was another reason why respondents turned to private health. If the doctor's appointment was not helpful, getting another appointment for a second opinion is very difficult.

*Unfortunately, I had to resort to paid consultations to find a gynecologist who really wanted to treat my pains and not make me feel guilty on top of everything*. (P1029, Galicia, YOB 1996)

*That primary care doctor who advised me to change my pad more often when I told him that my periods were very heavy. A private gynecologist diagnosed me with an endometrial polyp)*. (P1752, Comunidad Valenciana, YOB 1977)

In addition, some participants believed they would be better treated by a private gynecologist. This appears to be not an isolated belief, whether this is due to one's own experiences or from the testimonies of others.

*At the public health service, I'm afraid that I won't feel comfortable with the gynecologist, as I've heard countless stories of situations I don't want to go through*. (P1066, Extremadura, YOB 1998)

##### 3.3.5.3 The economic cost of being treated

The economic cost associated with all the diagnoses and treatments was present in responses. The financial cost was identified as a barrier to seeking solutions. The price of the private doctor and all the tests required when using private health, as well as the cost of prescribed contraceptive pills. Furthermore, the cost of other specialists like nutritionists or physiotherapists limited the respondents' options to potentially find a suitable treatment.

*To this day, the only treatment offered to me is the contraceptive pill, even though there are studies that affirm that nutrition and exercise are key elements in the treatment. Thus, the only solution left for me is to seek a specialized nutritionist and trainer and save up to be able to afford it*. (P879, Madrid, YOB 1995)

#### 3.3.6 A gender issue

Many women voiced the role they believed gender has in these interactions and the lack of research and solutions provided. It is perceived that not only women's pain is disregarded more often, but also women and their concerns are belittled and listened to less by doctors. Respondents expressed their impression that men and their sex-specific health issues do not face the same challenges when seeking medical attention, and their experiences are considered more trustworthy.

*Many women are neglected, if not the majority. We are not taken into consideration, and our symptoms are trivialized and diminished*. (P1741, Comunidad Valenciana, YOB 1978)

*The social view on menstruation today continues to be phallocentric and sexist. ...I am absolutely convinced that if men menstruated, there would be very substantial changes*. (P1347, Cataluña, YOB 1980)

## 4 Discussion

This study aims to examine the needs and challenges women encounter when seeking professional support, with the goal of identifying effective strategies to address these needs and ultimately improve their quality of life. The results demonstrate the relevance that menstrual disorders and discomforts have on the life of Spanish women and the challenges faced when seeking help for menstrual-related issues. Similar obstacles have been identified in other countries (17, 32–34). Different degrees of discomfort and severity of pain were indicated, from mild discomfort to disabling pain. The normalization of these symptoms, independent of their severity, contributes to the late diagnosis of underlying disorders with significant health implications. For example, endometriosis was apparent in our data, as well as the testimonies narrating the years of struggles to reach a diagnosis, in line with studies that established a delay of 4–11 years from first symptom onset to surgical diagnosis (35). Early and accurate diagnosis of conditions like endometriosis and adenomyosis is key to prevent long-term complications such as infertility and chronic pain (36). The difficulty in diagnosing these conditions, especially without advanced diagnostic tools such as ultrasound and MRI, underscores the need for increased awareness and education among healthcare providers (5).

Participants expressed concern that their treatments were focused on the symptoms without investigating possible underlying causes, an issue that has been previously highlighted (37). The perceived standardization of treatment for menstrual-related issues was flagged as a concern by many participants. The contraceptive pill was reported to be prescribed as a treatment for menstrual discomforts, including pain, irregular periods, excessive bleeding, etc. While some respondents reported positive improvements under the contraceptive pill treatment, others rejected the treatment for a range of reasons and signaled the absence of information provided about the potential effects and the lack of prior examination of such prescription. Furthermore, often, the contraceptive pill was perceived as the only alternative provided, and if participants rejected such treatment, they felt abandoned as often that was the only option. Similar findings were reported by previous studies where the options available for dysmenorrhea were to manipulate the cycle with hormonal contraception or get on with the pain (3).

Currently, the pharmacological treatment of dysmenorrhea includes nonsteroidal anti-inflammatory drugs (NSAIDs), such as ibuprofen; analgesic therapies, such as paracetamol; and hormonal therapies, such as contraception (38). However, the effectiveness of these treatments varies among individuals, as do the side effects (39). Therefore, a more personalized approach is needed, wherein therapies are adjusted to address individual patient responses (40). For refractory cases, surgical interventions or advanced options, such as gonadotropin-releasing hormone (GnRH) analogs, may be considered. However, these approaches require careful patient monitoring and informed decision-making (4).

Respondents often aspired to have more options and suggested that a more comprehensive approach to their diagnosis and treatment could increase their chances of finding a suitable treatment to improve their health. Their demands are sustained by several studies showing how other approaches, including lifestyle correction, exercise, physiotherapy for pelvic pain, nutrition, and micronutrients, can help improve menstrual function without relying solely on hormone therapy (2, 41–44).

The therapeutic itinerary analysis provided evidence that women faced difficulties related to the search for the diagnosis, consultations with specialized professionals, and failures in communication, reception, and care regarding menstrual health issues. Despite the high frequency of reported menstrual discomfort, a significant proportion of those who experienced monthly pain have never consulted a gynecologist. Many respondents found medical support insufficient, and the value of consulting a gynecologist has come into question, a perception not unique to the Spanish context. For example, Ní Chéileachair et al. (3) reported that consultations with medical professionals did not necessarily lead to a greater understanding of or relief from menstrual pain in their study on dysmenorrhea. The invalidation of their experiences, as well as the lack of alternatives to manage their symptoms, particularly their pain, might lead to the acceptance of these conditions. Furthermore, when shared, the perceived dismissal and invalidation might prevent others from seeking health care in the future. Some of the participants who were reluctant to visit the gynecologist were not due to their own previous negative experiences, but to the shared experiences of others. Despite this, data suggest that if given the possibility, women would follow up with specialists. For instance, in Spain, in 2021, there were 182,670 first visits to private gynecologists compared to a total of 310,761 recurrent visits. In contrast, the public system saw 14,053 first visits and 12,589 recurrent visits (23).

The relationship between menstrual pain and the frequency of medical consultations is well documented, with women experiencing severe dysmenorrhea tending to seek medical care more frequently due to the intensity of the pain and its impact on quality of life (1, 7). However, those with monthly discomforts do not necessarily visit the gynecologist more frequently. Instead, according to our data, individuals experiencing discomforts *some months* or *most months* are more likely to seek medical attention, highlighting the impact of perceived abnormalities on healthcare-seeking behavior. No significant statistical correlation was found between discomfort frequency and gynecologist visits, suggesting other influencing factors, such as healthcare accessibility and normalization of menstrual pain (30, 45). Menstrual literacy, which includes knowledge about the menstrual cycle and related conditions, is also known to influence the pursuit of medical care significantly. Thus, inadequate information and social stigma can inhibit women from seeking professional help (29, 30, 46).

Additionally, as our results show, the choice of healthcare provider influences the frequency of gynecological visits, as private healthcare users tend to have better access to specialized services and shorter waiting times, facilitating more appropriate management of menstrual discomforts (15). In the case of Spain, where public health is universal, the use of private gynecologists seems surprisingly frequent. This appears to be the result of several factors, e.g., the testimonies of negative experiences in gynecology in the public health system, the difficulty of getting an appointment, and the lack of solutions provided. Particularly, the difficulty of accessing gynecologists in the public system may be a crucial factor. It could explain, for instance, why women experiencing frequent menstrual discomforts tend to consult gynecologists more frequently in private healthcare settings compared to public ones. Furthermore, over half of gynecological consultations (52.7%) in public healthcare during 2021 were first-time visits, indicating that most people only use the system once. This lack of follow-up suggests ongoing care problems, potentially hindering gynecological health management in the public sector. It might also highlight a systemic issue, whereby pressures on the healthcare system result in the deprioritization of non-urgent or non-malignant issues, without due consideration for the impact on functioning or quality of life (17).

As expected, socioeconomic factors, including income levels, impact the choice between private and public healthcare. Women with higher incomes are more likely to opt for private healthcare, often resulting in more timely and appropriate care (47). While not everyone who can afford private healthcare will choose it over public healthcare, those who prefer private healthcare can only access it if they can afford it. This economic barrier becomes a source of inequality, especially when obtaining support from public health services is challenging.

When considering the decade of birth, data show a decrease in annual visits from those born in the 1950s compared to those born in the 2000s and an increase in those never visiting a gynecologist. This might be influenced by a variety of factors. Different generations present different behaviors regarding access to information, and younger generations are increasingly turning to social media for health information. A study involving 42,000 individuals across 40 countries found that 44% of Gen Z (born 1997–2004) and 41% of Millennials (born 1981–1996) rely on social media for health information they consider useful and trustworthy (48).

Furthermore, there appears to be a generational shift in what is deemed trustworthy: 17% of Gen Z and 14% of Millennials view social media, but not their doctors, as a source of reliable information. In contrast, only 10% of Gen X (born 1965–1980) and 5% of Baby Boomers (born 1946–1964) share this view (48). It remains unclear which comes first: do individuals turn to social media for health information because doctors have failed to provide adequate solutions, or do they avoid seeking medical advice because they trust the information found on social media and feel they do not need expert advice? This should be considered when addressing different generations regarding health information.

This study reveals interesting trends related to menstrual discomforts and gynecological consultations. 70.9% of respondents experience menstrual discomfort monthly or most months, with pain and abdominal swelling being the most common. Interestingly, the frequency of reported discomforts has increased over the decades, indicating either a rise in these symptoms and/or greater awareness. The authors are more inclined to the former explanation. It might be the case of different factors that translate into recent generations speaking up more and challenging the normalization of their health issues, and feminism may have a role in this.

Feminism has spent a great deal of effort fighting for equality in health and criticizing the use of health as a tool of dominance against women. At the end of the 19th century, there was a strong fight for women's access to medicine. During this period, medical discourse that attributed women's discomfort, weakness, and illness to their biology was also challenged and it was argued that social aspects such as the restriction of women's freedom to develop intellectually, the exploitation of working-class women, or the inactivity of bourgeois women were the true causes of the ailments that were biologically attributed to them (49). In the 20^*th*^ century, as women entered universities and the professional healthcare field, numerous studies highlighted the disparities in medical care received by men and women. These studies also emphasized that women's health needs extend beyond reproductive concerns (49). Moving into the 21^*st*^ century, science, particularly health science, has been scrutinized from a feminist perspective, revealing significant scientific and clinical biases (50).

Each generation is exposed to the social and historical context of their time. Feminist movements have strengthened and been more present in Spain in the last decades (51) and younger generations might have been influenced by the reflections and demands of contemporary feminism, which highlights the structural inequalities that limit women's agency and rights, including demanding medical attention for health issues related to their sexual and reproductive health.

Some respondents considered being a woman an important factor that conditioned the attention received from the doctors, from not being listened to, to the invalidation or normalization of their pain. There is an extensive literature documenting similar cases (16–19). Similar occurrences happening in different countries highlight an issue that transcends a specific social context. Nogueiras García (49) reflection exposes a systemic problem: “*The imaginary created by the patriarchy about the health of women and their natural proclivity to the development of physical and emotional discomforts underlies the objectivity of science and continues to be applied in current health practices*.” The disparity caused by the gender pain gap, already introduced in this article, can lead to misdiagnosis, delayed treatment, and poorer health outcomes for women.

Traditional health concepts often overlook women's diverse needs, highlighting the need for inclusive, gender-sensitive healthcare (52, 53). Applying feminist theory to medical education has the potential to create structural change (54), and address health inequities by examining gender, disadvantage, and power distribution in public health (55, 56).

## 5 Strengths and limitations

While attempts were made to reach all sociodemographic groups, this study likely did not recruit enough members of vulnerable groups and groups at risk of exclusion to be representative of their experiences. Furthermore, the average higher education level is high in the sample, with a notable predominance of university-educated individuals. Views captured here pertain to voluntarily reported individuals, which might introduce bias in the sample.

Limitations notwithstanding, the current study makes a significant contribution through the data gathered and its emphasis on the experiential aspects of healthcare seeking help for issues related to menstruation, to the best of our knowledge, being the first study of this kind in Spain. The strengths of the present study include the rigorous analysis of an understudied topic and the considerable sample used to gather information. The sample reached representativeness in all regions of Spain and has a good representation of a range of ages. This study provides important insight into the experience of seeking care for menstrual issues in Spain. Further research is needed to delve into the doctor-patient interaction in this context. For instance, studying the challenges doctors face when trying to diagnose potential issues and analyzing their experience.

## 6 Recommendations

The inclusion of gender perspective in medical training and healthcare professionals might be a strategic starting point to address integrated gender biases in medicine (54).

Healthcare professionals need to be adequately educated and equipped to recognize, diagnose, and treat dysmenorrhea and its potential underlying pathologies. This should include targeted training and resources to enhance their capacity for early detection and comprehensive management, ultimately improving patient outcomes.

Campaigns to promote seeking medical attention when pain interferes with daily life might be beneficial in the fight against the gendered views of women's pain. In addition, efforts should be placed into building trust in the specialists from the public system and increase the help-seeking rating.

## 7 Conclusions

The frequency of medical dismissal reported by participants signals the need for improvements in the Spanish medical care system to ensure that concerns related to menstruation are taken seriously and managed appropriately.

Recognizing the patients' menstrual experiences is essential for doctors. It informs the development of strategies to encourage healthcare-seeking, which can lead to improved management of dysmenorrhea and potentially earlier diagnosis of underlying pathological conditions.

This paper provides a baseline that demonstrates the challenges found when seeking medical support for menstrual-related disorders in Spain. This information can be used to inform decisions and shape measures to improve menstrual experience and menstrual health. New research could use this data as a point of reference for further exploration. Future research might investigate the dynamics of these interactions (doctors and women), identifying potential barriers to effective communication and empathy.

## Data Availability

The raw data supporting the conclusions of this article will be made available by the authors, without undue reservation.

## References

[1] Iacovides S Avidon I Baker FC. What we know about primary dysmenorrhea today: a critical review. Hum Reprod Update. (2015) 21:762–78. 10.1093/humupd/dmv039 26346058

[2] Brown N Martin D Waldron M Bruinvels G Farrant L Fairchild R. Nutritional practices to manage menstrual cycle-related symptoms: a systematic review. Nutr Res Rev. (2023) 37:352–75. 10.1017/S0954422423000227 37746736

[3] Ní Chéileachair F McGuire BE Durand H. Coping with dysmenorrhea: a qualitative analysis of period pain management among students who menstruate. BMC Womens Health. (2022) 22:1–11. 10.1186/s12905-022-01988-4 36199106 PMC9533282

[4] Martire FG Piccione E Exacoustos C Zupi E. Endometriosis and adolescence: the impact of dysmenorrhea. J Clin Med. (2023) 12:5624. 10.3390/jcm12175624 37685691 PMC10488856

[5] Martire FG d'Abate C Schettini G Cimino G Ginetti A Colombi I. Adenomyosis and adolescence: a challenging diagnosis and complex management. Diagnostics. (2024) 14:2344. 10.3390/diagnostics14212344 39518312 PMC11544982

[6] Fernández Macedo SA Agüero JJ Salas GB Fernandez Tapia SB Rossel EC. Semiologic differences and primary dysmenorrhea. Heliyon. (2023) 9:e19489. 10.1016/j.heliyon.2023.e19489 37810005 PMC10558588

[7] Dawood MY. Nonsteroidal anti-inflammatory drugs and changing attitudes toward dysmenorrhea. Am J Med. (1988) 84:23–9. 10.1016/0002-9343(88)90473-1 3287908

[8] Altman G Cain KC Motzer S Jarrett M Burr R Heitkemper M. Increased symptoms in female IBS patients with dysmenorrhea and PMS. Gastroenterol Nurs. (2006) 29:4–11. 10.1097/00001610-200601000-00002 16552294

[9] Chen CX Draucker CB Carpenter JS. What women say about their dysmenorrhea: a qualitative thematic analysis. BMC Womens Health. (2018) 18:1–8. 10.1186/s12905-018-0538-8 29499683 PMC5833075

[10] Chen CX Kwekkeboom KL Ward SE. Beliefs about dysmenorrhea and their relationship to self-management. Res Nurs Health. (2016) 39:263–76. 10.1002/nur.21726 27177093 PMC7509811

[11] Leon-Larios F Silva-Reus I Puente Martínez MJ Renuncio Roba A Ibeas Martínez E Lahoz Pascual I. Influence of menstrual pain and symptoms on activities of daily living and work absenteeism: a cross-sectional study. Reprod Health. (2024) 21:1–10. 10.1186/s12978-024-01757-6 38374080 PMC10875820

[12] Vannuccini S Rossi E Cassioli E Cirone D Castellini G Ricca V. Menstrual Distress Questionnaire (MEDI-Q): a new tool to assess menstruation-related distress. Reprod Biomed Online. (2021) 43:1107–16. 10.1016/j.rbmo.2021.08.029 34753680

[13] Vincent K Warnaby C Stagg CJ Moore J Kennedy S Tracey I. Dysmenorrhoea is associated with central changes in otherwise healthy women. Pain. (2011) 152:1966–75. 10.1016/j.pain.2011.03.029 21524851

[14] Silva LFd Cursino EG Brandão EdS Góes FGB Depiant JRB Silva ea L J da. The therapeutic itinerary of health workers diagnosed with COVID-19. Rev Latino-Am Enferm. (2021) 29:e3413. 10.1590/1518-8345.4691.3413 33852685 PMC8040779

[15] Casacio GDdM Ferrari RAP Zilly A Silva RMMd. Therapeutic itinerary of children with special health care needs: analysis guided by care systems. Rev Gauch Enferm. (2022) 43:1–12. 10.1590/1983-1447.2022.20210115.pt 35613243

[16] Kolmes SK Boerstler KR. Is there a gender self-advocacy gap? An empiric investigation into the gender pain gap. J Bioeth Inq. (2020) 17:383–93. 10.1007/s11673-020-09993-8 32728800

[17] Windrim EB McGuire BE Durand H. Women's experiences of seeking healthcare for abdominal pain in Ireland: a qualitative study. BMC Womens Health. (2024) 24:1–13. 10.1186/s12905-024-02995-3 38454395 PMC10921746

[18] Samulowitz A Gremyr I Eriksson E Hensing G. Brave Men and Emotional Women: a theory-guided literature review on gender bias in health care and gendered norms towards patients with chronic pain. Pain Res Manag. (2018) 2018:6358624. 10.1155/2018/6358624 29682130 PMC5845507

[19] Colet C Amador TA Heineck I. Therapeutic itinerary: trajectory for resolution of adverse events of patients using warfarin in Southern Brazil. Braz J Pharm Sci. (2018) 54:1–8. 10.1590/s2175-97902018000317738

[20] Secretaría General de Salud Digital Información e Innovación del Sistema Nacional de Salud. (2023). Sistema de Información sobre listas de espera en el Sistema Nacional de Salud. Ministerio de Sanidad. Available at: https://administracion.gob.es/ (accessed October 25, 2024).

[21] OECD. Health status: Health care resources by provider - *obstetricians and gynaecologists*. OECD. (2023).

[22] INE. Demographic statistics. (2023).

[23] Sanidad MDE. Estadística de centros de atención especializada. Año (2021). Available at: https://www.sanidad.gob.es/estadEstudios/estadisticas/sisInfSanSNS/tablasEstadisticas/InfAnualSNS2020_21/INFORME_ANUAL_2020_21.pdf (accessed October 21, 2024).

[24] Sánchez López S Barrington DJ Poveda Bautista R. Spanish menstrual literacy and experiences of menstruation. BMC Women's Health. (2023) 23:161. 10.1186/s12905-023-02293-4 37016318 PMC10074887

[25] Corbetta P. Metodología y técnicas de investigación social. Madrid, Spain: McGraw-Hill Interamericana de Espa. (2010).

[26] O'Brien BC Harris IB Beckman TJ Reed DA Cook DA. Standards for reporting qualitative research: a synthesis of recommendations. Acad Med. (2014) 89:1245–51. 10.1097/ACM.0000000000000388 24979285

[27] EQUATOR Network. Enhancing the QUAlity and Transparency of health Research. (2022). Available at: https://www.equator-network.org/ (accessed August 23, 2024).

[28] Hennegan J Brooks DJ Schwab KJ Melendez-Torres GJ. Measurement in the study of menstrual health and hygiene: a systematic review and audit. PLoS ONE. (2020) 15:e0232935. 10.1371/journal.pone.0232935 32497117 PMC7272008

[29] Hennegan J Nansubuga A Akullo A Smith C Schwab KJ. The Menstrual Practices Questionnaire (MPQ): development, elaboration, and implications for future research. Glob Health Action. (2020) 13:1829402. 10.1080/16549716.2020.1829402 33052077 PMC7594862

[30] Phillips-Howard PA Caruso B Torondel B Zulaika G Sahin M Sommer M. Menstrual hygiene management among adolescent schoolgirls in low- and middle-income countries: research priorities. Glob Health Action. (2016) 9:33032. 10.3402/gha.v9.33032 27938648 PMC5148805

[31] Olmos-Vega FM Stalmeijer RE Varpio L Kahlke R. A practical guide to reflexivity in qualitative research: AMEE Guide No. 149 Med Teach. (2023) 45:241–51. 10.1080/0142159X.2022.2057287 35389310

[32] Barrington DJ Robinson HJ Wilson E Hennegan J. Experiences of menstruation in high income countries: a systematic review, qualitative evidence synthesis and comparison to low-and middle-income countries. PLoS ONE. (2021) 16:e0255001. 10.1371/journal.pone.0255001 34288971 PMC8294489

[33] Matías-González Y Sánchez-Galarza AN Flores-Caldera I Rivera-Segarra E. “Es que tú eres una changa”: stigma experiences among Latina women living with endometriosis. J Psychosom Obstet Gynecol. (2021) 42:67–74. 10.1080/0167482X.2020.1822807 32964770 PMC8893272

[34] Maulenkul T Kuandyk A Makhadiyeva D Dautova A Terzic M Oshibayeva A. Understanding the impact of endometriosis on women's life: an integrative review of systematic reviews. BMC Women's Health. (2024) 24:524. 10.1186/s12905-024-03369-5 39300399 PMC11411992

[35] Agarwal SK Chapron C Giudice LC Laufer MR Leyland N Missmer S. Clinical diagnosis of endometriosis: a call to action. Am J Obstet Gynecol. (2019) 220:354.e1–354.e12. 10.1016/j.ajog.2018.12.039 30625295

[36] Griffiths MJ Horne AW Gibson DA Roberts N Saunders PTK. Endometriosis: recent advances that could accelerate diagnosis and improve care. Trends Mol Med. (2024) 30:875–89. 10.1016/j.molmed.2024.06.008 38991858

[37] Armour M Parry K Al-Dabbas MA Curry C Holmes K. MacMillan F. Self-care strategies and sources of knowledge on menstruation in 12,526 young women with dysmenorrhea: a systematic review and meta-analysis. PLoS ONE. (2019) 14:1–18. 10.1371/journal.pone.0220103 31339951 PMC6655766

[38] Kirsch E Rahman S Kerolus K Hasan R Kowalska DB Desai A. Dysmenorrhea, a narrative review of therapeutic options. J Pain Res. (2024) 17:2657–66. 10.2147/JPR.S459584 39161419 PMC11332412

[39] Chen CX Carpenter JS LaPradd M Ofner S Fortenberry JD. Perceived ineffectiveness of pharmacological treatments for dysmenorrhea. J Women's Health. (2021) 30:1334–43. 10.1089/jwh.2020.8581 33026968 PMC8558084

[40] MacGregor B Allaire C Bedaiwy MA Yong PJ Bougie O. Disease burden of dysmenorrhea: impact on life course potential. Int J Women's Health. (2023) 15:499–509. 10.2147/IJWH.S380006 37033122 PMC10081671

[41] Tremback-Ball A Hammond E Applegate A Caldwell E Witmer H. Effectiveness of physical therapy interventions for women with dysmenorrhea: a systematic review. J Women's Health Phys Ther. (2023) 47:3–18. 10.1097/JWH.0000000000000258

[42] Kuznetsova IV. Non hormonal management options for menstrual cycle irregularities. Med Counc. (2019) 12:16–27. 10.21518/2079-701X-2019-13-16-27

[43] Cronk N Zweig A Deane K. Is exercise an effective treatment for dysmenorrhea? Evid Based Pract. (2021) 24:32–3. 10.1097/EBP.0000000000001138

[44] Berghmans B. Physiotherapy for pelvic pain and female sexual dysfunction: an untapped resource. Int Urogynecol J. (2018) 29:631–8. 10.1007/s00192-017-3536-8 29318334 PMC5913379

[45] Armour M Parry K Manohar N Holmes K Ferfolja T Curry C. The prevalence and academic impact of dysmenorrhea in 21,573 young women: a systematic review and meta-analysis. J Women's Health. (2019) 28:1161–71. 10.1089/jwh.2018.7615 31170024

[46] Sommer M Hirsch JS Nathanson C Parker RG. Comfortably, safely, and without shame: defining menstrual hygiene management as a public health issue. Am J Public Health. (2015) 105:1302–11. 10.2105/AJPH.2014.302525 25973831 PMC4463372

[47] Courtemanche C Marton J Ukert B Yelowitz A Zapata D Fazlul I. The three-year impact of the Affordable Care Act on disparities in insurance coverage. Health Serv Res. (2019) 54:307–16. 10.1111/1475-6773.13077 30378119 PMC6341207

[48] Impact RE. Empowering healthy communities and individuals: Removing barriers through health literacy. (2024). Available at: https://impact.economist.com/projects/health-inclusivity-index/articles/empowering-healthy-communities-and-individuals?utm_source=linkedin&utm_medium=paid-ei-social&utm_campaign=Article-3-CXO&utm_content=native_size/&li_fat_id=51a2d2e6-d880-4bb1-bfbb-0d252a59fbe8 (accessed October 21, 2024).

[49] Nogueiras García B. La salud en la teoría feminista. Atlánticas - *Rev Int Estud Fem*. (2019) 4:10–31. 10.17979/arief.2019.4.1.5404

[50] Dauder G Sedeño SP Las E. Las mentiras cient-ficas sobre las mujeres. ArtefaCToS - *Rev Estud la Cienc y la Tecnol*. (2018) 7:211–4.

[51] Galdón Corbella C. Cosmovisiones feministas en clave generacional. Del movimiento 15M a la Huelga Feminista del 8M. Encrucijadas - *Rev Crítica Ciencias Soc*. (2018) 16:v1602. Available at: https://recyt.fecyt.es/index.php/encrucijadas/article/view/79175 (accessed October 20, 2024).

[52] Hockey J. Women and Health. In:Robinson V Richardson D, editors. Introducing women's studies: Feminist theory and practice. London: Macmillan Education UK (1997). p. 282–302. 10.1007/978-1-349-25726-3_13

[53] Eduarda de Araújo Torres A Patrícia de Oliveira A Concei cão de Assis J Carolina Salustino dos Santos M. Natural gynecology: theory and practice. Heal Soc. (2023) 3:53–9. 10.51249/hs.v3i03.1242

[54] Sharma M. Applying feminist theory to medical education. Lancet. (2019) 393:570–8. 10.1016/S0140-6736(18)32595-9 30739692

[55] Rogers WA. Feminism and public health ethics. J Med Ethics. (2006) 32:351–4. 10.1136/jme.2005.013466 16731735 PMC2563367

[56] Shai A Koffler S Hashiloni-Dolev Y. Feminism, gender medicine and beyond: a feminist analysis of “gender medicine.” *Int J Equity Health*. (2021) 20:1–11. 10.1186/s12939-021-01511-5 34344374 PMC8330093

