# Supplementary material for: A mixed method study of menstrual health in Spain: pain, disorders, and the journey for health
Source: Front Public Health. 2025 Feb 25;13:1517302. doi: 10.3389/fpubh.2025.1517302 (PMC11893987; doi:10.3389/fpubh.2025.1517302)
Supplement: Supplementary file 1 [file Data_Sheet_1.pdf]

Supplementary Material

1 STANDARDS FOR REPORTING QUALITATIVE RESEARCH: A SYNTHESIS OF RECOMMENDATIONS

No.	Topic	Item	Section in the paper
	**Title and Abstract**		
S1	Title	Concise description of the nature and topic of the study. Identifying the study as qualitative or indicating the approach (e.g., ethnography, grounded theory) or data collection methods (e.g., interview, focus group) is recommended.	Navigating Menstrual Health in Spain: Pain, Disorders, and the Search for Medical Care from a Mixed Methodological Approach
S2	Abstract	Summary of key elements of the study using the abstract format of the intended publication; typically includes background, purpose, methods, results, and conclusions.	Abstract
	**Introduction**		
S3	Problem formulation	Description and significance of the problem/phenomenon studied; review of relevant theory and empirical work; problem statement.	Introduction
S4	Purpose or research question	Purpose of the study and specific objectives or questions.	Introduction
	**Methods**		
S5	Qualitative approach and research paradigm	Qualitative approach (e.g., ethnography, grounded theory, case study, phenomenology, narrative research) and guiding theory if appropriate; identifying the research paradigm (e.g., postpositivist, constructivist/interpretivist) is also recommended; rationale.	Materials and Methods. Data Analysis
S6	Researcher characteristics and reflexivity	Researchers' characteristics that may influence the research, including personal attributes, qualifications/experience, relationship with participants, assumptions, and/or presuppositions; potential or actual interaction between researchers' characteristics and the research questions, approach, methods, results, and/or transferability.	Materials and Methods. Reflexivity

Table S1. Summary of Qualitative Study Reporting Standards

No.	Topic	Item	Section in the paper
S7	Context	Setting/site and salient contextual factors; rationale.	
S8	Sampling strategy	How and why research participants, documents, or events were selected; criteria for deciding when no further sampling was necessary (e.g., sampling saturation); rationale.	Materials and Methods. Participants and recruitment
S9	Ethical issues pertaining to human subjects	Documentation of approval by an appropriate ethics review board and participant consent, or explanation for lack thereof; other confidentiality and data security issues.	Materials and Methods. Ethical considerations
S10	Data collection methods	Types of data collected; details of data collection procedures including (as appropriate) start and stop dates of data collection and analysis, iterative process, triangulation.	Materials and Methods. Participants and recruitment
S11	Data collection instruments and technologies	Description of instruments (e.g., interview guides, observation rubrics) and devices (e.g., audio recorders) used for data collection; if/how the instruments changed over the course of the study.	Materials and Methods. Data collection instruments
S12	Units of study	Number and relevant characteristics of participants, documents, or events included in the study; level of participation (could be reported in results).	Materials and Methods. Participants and recruitment
S13	Data processing	Processes for data coding and processing; rationale.	Figure 2. Qualitative Study
S14	Data analysis	Process by which inferences, themes, etc. were identified and developed, including researchers involved in data analysis; usually references a specific paradigm or approach; rationale.	Materials and Methods. Data Analysis
S15	Techniques to enhance trustworthiness	Techniques to enhance the quality of data (e.g., member checking, audit trail, triangulation); rationale.	N/A

Table S2. Summary of Qualitative Study Reporting Standards

No.	Topic	Item	Section in the paper
	**Discussion**		
S16	Synthesis and interpretation	Main findings (typically reported as themes, categories, concepts, or in the form of a model); and their meanings/significance.	Figure 2. Qualitative Study
S17	Links to empirical data	Short quotations, descriptions of events, participant observations, etc., to illustrate major findings; typically reported in results.	Results. Qualitative studies. Multiple quotations and participants comments.
	**Results / findings**		
S18	Integration with prior work, implications, transferability, and contribution(s) to the field	Integration with prior research, implications for practice or policy, study limitations, potential for transferability, and implications for future research.	Discussion. Study limitations: Discussion. Strengths and limitations
S19	Limitations	Study limitations related to design, data analysis, and/or transferability.	Discussion. Strengths and limitations
	**Other**		
S20	Conflicts of interest	Potential sources of influence or perceived influence on study conduct and conclusions; how these were managed.	Declarations. Competing interest
S21	Funding	Sources of funding and other support; role of funders in data collection, interpretation, and reporting.	Declarations. Funding

Table S3. Summary of Qualitative Study Reporting Standards
